# Spatial ecology of territorial populations

**DOI:** 10.1073/pnas.1911570116

**Published:** 2019-08-21

**Authors:** Benjamin G. Weiner, Anna Posfai, Ned S. Wingreen

**Affiliations:** ^a^Department of Physics, Princeton University, Princeton, NJ 08544;; ^b^Simons Center for Quantitative Biology, Cold Spring Harbor Laboratory, Cold Spring Harbor, NY 11724;; ^c^Lewis–Sigler Institute for Integrative Genomics, Princeton University, Princeton, NJ 08544

**Keywords:** spatial ecology, biodiversity, trade-offs, microbial ecology, modeling

## Abstract

Many ecosystems, from vegetation to biofilms, are composed of territorial populations that compete for both nutrients and physical space. What are the implications of such spatial organization for biodiversity? To address this question, we developed and analyzed a model of territorial resource competition. In the model, all species obey trade-offs inspired by biophysical constraints on metabolism; the species occupy nonoverlapping territories, while nutrients diffuse in space. We find that the nutrient diffusion time is an important control parameter for both biodiversity and the timescale of population dynamics. Interestingly, fast nutrient diffusion allows the populations of some species to fluctuate to zero, leading to extinctions. Moreover, territorial competition spontaneously gives rise to both multistability and the Allee effect (in which a minimum population is required for survival), so that small perturbations can have major ecological effects. While the assumption of trade-offs allows for the coexistence of more species than the number of nutrients—thus violating the principle of competitive exclusion—overall biodiversity is curbed by the domination of “oligotroph” species. Importantly, in contrast to well-mixed models, spatial structure renders diversity robust to inequalities in metabolic trade-offs. Our results suggest that territorial ecosystems can display high biodiversity and rich dynamics simply due to competition for resources in a spatial community.

## Significance

All organisms live in spatial communities. In many cases, such as vegetation or bacterial biofilms, dense surface-bound populations compete for both resources and physical space. How do these territorial interactions impact ecosystem behavior and biodiversity? We study a theoretical model of territorial resource competition with trade-offs and show that many features of real ecosystems emerge naturally, including slow population dynamics that render community composition susceptible to demographic and other noise. We also observe alternate steady states, including the Allee effect in which survival requires a minimum population. Importantly, we demonstrate that biodiversity occurs robustly and can arise in territorial communities simply due to competition for resources.

Living things exist not in isolation but in communities, many of which are strikingly diverse. Tropical rainforests can have more than 300 tree species in a single hectare (1), and it has been estimated that 1 g of soil contains 2,000–30,000+ distinct microbial genomes (2, 3). Understanding the relationship between biodiversity and the environment remains a major challenge, particularly in light of the competitive exclusion principle: In simple models of resource competition, no more species can coexist indefinitely than the number of limiting resources (4, 5). In modern niche theory, competitive exclusion is circumvented by mechanisms which reduce niche overlaps and/or intrinsic fitness differences (6, 7), suggesting that trade-offs may play an important role in the maintenance of biodiversity. Intriguingly, diversity beyond the competitive-exclusion limit was recently demonstrated in a resource-competition model with a well-mixed environment and exact metabolic trade-offs (8). However, many ecosystems are spatially structured, and metabolic trade-offs are unlikely to be exact. While some spatial structure is externally imposed, it also arises from the capacity of organisms to shape their environment. How does self-generated spatial structure, along with realistic metabolic constraints, impact diversity?

Various studies have clarified how intrinsic environmental heterogeneity (e.g., an external resource gradient) fosters biodiversity by creating spatial niches (9–13). Others have demonstrated that migration between low-diversity local environments can lead to “metacommunities” with high global diversity (14–19). But how is diversity impacted by local spatial structure? Recent models suggest that spatial environments without intrinsic heterogeneity can support higher diversity than the well-mixed case (20–25), although the effect depends on the interactions and details of spatial structure (26, 27). In these models, competition follows phenomenological interaction rules. In some cases, trade-offs have been invoked to limit fitness differences (21) and penalize niche overlap (25), but did not otherwise structure the spatial interactions. All these models allow coexistence when the combination of spatial segregation and local interactions weakens interspecific competition relative to intraspecifc competition. However, it remains unclear how such interactions relate to concrete biophysical processes.

Here, we study biodiversity in a model where species interact through spatial resource competition. We specifically consider surface-associated populations which exclude each other as they compete for territory. This is an appropriate description for biofilms, vegetation, and marine ecosystems like mussels (28) or coral (29), in contrast with models that represent populations as overlapping densities and better describe motile or planktonic populations (9, 30). The well-mixed environment is an explicit limit of our model, so we are able to isolate the unique effects of spatial structure.

We find that, contrary to expectations, introducing population territories into a model with metabolic trade-offs reduces biodiversity relative to the well-mixed case. Extinctions occur over a new timescale inversely related to the nutrient mixing time. Spatial structure also leads to the emergence of multiple steady states and the Allee effect, so that small perturbations may have drastic consequences. Finally, we find that overall biodiversity is curbed by the domination of “oligotroph” species but is robust to inequalities in metabolic trade-offs.

## Results

### Model.

We developed a model of territorial populations competing for diffusing resources to clarify the relationship between spatial structure, metabolic trade-offs, and biodiversity. The model is spatially explicit and relates the mechanistic dynamics of competition to parameters with clear biological meaning. Crucially, competing populations are not interpenetrating, so populations are competing for both nutrients and territory.

**Fig. 1.**
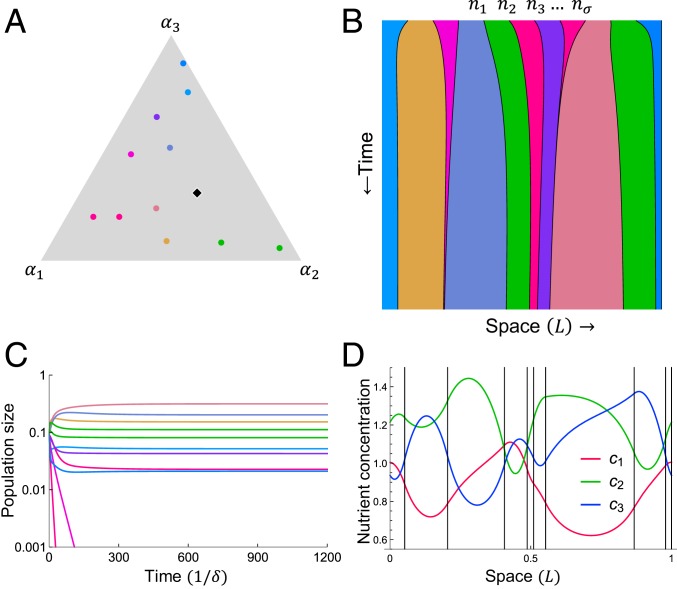
A model with spatial structure and metabolic trade-offs supports more species than expected from the principle of competitive exclusion. Example with 3 nutrients and 11 species starting with equal populations is shown. (*A*) Each species uptakes nutrients according to its enzyme-allocation strategy $\left(\alpha_{\sigma 1},\alpha_{\sigma 2},\alpha_{\sigma 3}\right)$. Because strategies satisfy the budget constraint $\sum_{i}\alpha_{\sigma i}=E$, each can be represented as a point on a triangle in strategy space. The nutrient supply $\vec{s}=\left(E/S\right)\vec{S}=\left(0.25,0.45,0.3\right)$ is represented as a black diamond. Colors correspond to strategies and are consistent throughout the figure. (*B*) Each species occupies a fraction of a 1-dimensional space (a ring) and has a corresponding time-dependent population size $n_{\sigma }\left(t\right)$. Here, the nutrient diffusion time $\tau_{D}$ is 400. (*C*) Population dynamics from *A*. Nine species coexist on 3 nutrients. (*D*) Concentrations of the 3 nutrients at steady state (vertical black lines denote boundaries between populations).

**Fig. 2.**
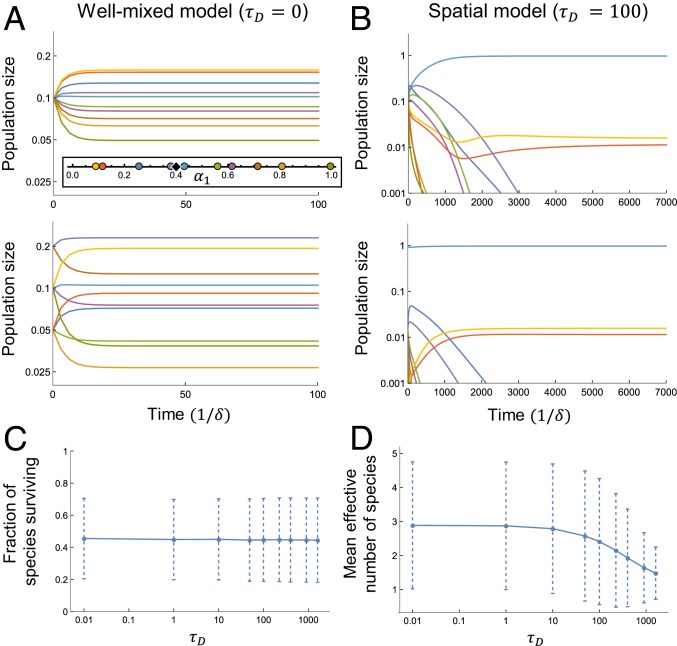
Spatial structure reduces diversity compared to the well-mixed limit of instantaneous nutrient diffusion. (*A*, *Upper*) A well-mixed population of 10 species with equal initial populations competing for 2 nutrients. All 10 coexist at steady state. (*A*, *Lower*) Same as *A*, *Upper*, but with different initial populations. The community reaches a new steady state. (*A*, *Upper*, *Inset*) Strategies ${\vec{\alpha }}_{\sigma }$ and resource supply $\vec{s}=\left(0.4,0.6\right)$. (*B*, *Upper*) Same species and nutrient supply as *A*, but in a spatial environment with nonzero nutrient diffusion time. Only 3 species survive. (*B*, *Lower*) Same as *B*, *Upper*, but with different initial populations. The community reaches the same steady state. (*C*) Fraction of initial species coexisting at steady state with $\vec{s}=\left(0.4,0.6\right)$; a population is considered extinct if $n_{\sigma }/L<10^{-6}$ (mean ± SD for 400 random sets of 10 strategies). The well-mixed model has survival fraction 0.99±0.08. (*D*) Effective number of species M at steady state (mean ± SD for same strategies as *C*).

Specifically, we consider m species competing for p nutrients in a 1-dimensional space of size L with periodic boundary conditions (a ring). The rate of supply of nutrients is specified by the supply vector $\vec{S}=\left(S_{1},S_{2}\ldots S_{p}\right)$ such that $\sum_{i}S_{i}=S$, where S is the total nutrient supply rate in units of concentration/time. The nutrient supply is spatially uniform, so there is no external environmental heterogeneity. Each species σ∈[1…m] is defined by its metabolic strategy ${\vec{\alpha }}_{\sigma }=\left(\alpha_{\sigma 1},\alpha_{\sigma 2}\ldots \alpha_{\sigma p}\right)$, which specifies the proportion of its metabolic resources (e.g., enzymes) it allocates to the consumption of each nutrient. Metabolic trade-offs are implemented via a constraint on the enzyme budget—namely, $\sum_{i}\alpha_{\sigma i}=E$ for all species (except where noted). Metabolic strategies and the supply can be represented as points on a simplex of dimension p−1 (see Figs. 1*A* and 2 *A*, *Inset*, for example). Each species occupies a segment of the ring corresponding to its population $n_{\sigma }$, so that $n_{\sigma }$ is a length and σ=1…m specifies a spatial ordering. For example, the population with strategy ${\vec{\alpha }}_{2}$ occupies the segment of the ring between populations with strategies ${\vec{\alpha }}_{1}$ and ${\vec{\alpha }}_{3}$. Populations never overlap, so the total population satisfies $\sum_{\sigma }n_{\sigma }=L$. Fig. 1 *B* and *C* shows an example of the time evolution of one such spatial community consisting of 11 species competing for 3 nutrients.

While the supply of nutrients is spatially uniform, the local rate of nutrient consumption depends on the metabolic strategy of the local species, and nutrients diffuse in space. We study the regime where population growth is nutrient-limited, so the rate of uptake of each nutrient is linear in its concentration. Thus, within each region occupied by a single species σ, the nutrient concentrations $c_{\sigma i}$ obey

$$\frac{\partial c_{\sigma i}}{\partial t}=S_{i}-\alpha_{\sigma i}c_{\sigma i}+D\frac{\partial^{2}c_{\sigma i}}{\partial x^{2}},$$ [1]

where D is the diffusion coefficient for all nutrients. As nutrient processing is generally much faster than growth, we assume a separation of timescales, such that nutrient concentrations equilibrate before populations change. Then, $\frac{\partial c}{\partial t}=0$, and

$$c_{\sigma i}\left(x\right)=\frac{S_{i}}{\alpha_{\sigma i}}+A_{\sigma i}\exp\left(x\sqrt{\frac{\alpha_{\sigma i}}{D}}\right)+B_{\sigma i}\exp\left(-x\sqrt{\frac{\alpha_{\sigma i}}{D}}\right).$$ [2]

The constants of integration $A_{\sigma i}$ and $B_{\sigma i}$ are fixed by the physical requirement that $c_{i}\left(x\right)$ be continuous and differentiable at the population boundaries. Fig. 1*D* shows the concentrations of the 3 nutrients after the populations shown in Fig. 1 *B* and *C* has reached steady state. The competitors transform the uniform nutrient supply into a complex spatial environment by depleting their preferred nutrients while allowing other nutrients diffuse to their neighbors.

The populations change in time according to

$$\frac{dn_{\sigma }}{dt}=\underset{i}{\sum }\alpha_{\sigma i}\left(v\int_{0}^{n_{\sigma }}\;c_{i}\left(x\right)\;dx\right)-\delta n_{\sigma },$$ [3]

where δ is the death rate and the integral is taken over the territory occupied by species σ. v is a length that converts nutrients to territory growth. The total population remains fixed at L, corresponding to competition for a share of a fixed total territory. This implies $\sum_{\sigma }{\dot{n}}_{\sigma }=0$, which requires δ=vS—i.e., the death rate matches the nutrient value of the total supply rate. We choose units of time and concentration such that v=1 and S=1, without loss of generality. In the example shown in Fig. 1 *B* and *C*, 9 species coexist, far exceeding the 3-species limit set by competitive exclusion.

The spatial nutrient environment influences the population dynamics via the dimensionless diffusion time $\tau_{D}\equiv L^{2}E/D$, which is the time for nutrients to diffuse a distance L relative to the uptake time. Competitors interact only through the nutrient environment, so when nutrients diffuse instantaneously ($\tau_{D}=0$), the spatial dynamics reduce to the well-mixed dynamics. (See *SI Appendix* for the $\tau_{D}\to 0$ expansion.)

### Biodiversity.

How does territorial spatial structure influence biodiversity? As an illustrative example, we consider 10 species competing for 2 resources. The simplex in Fig. 2 *A*, *Inset* shows how each of the strategies (colored dots) and the nutrient supply (diamond) divide between the 2 nutrients. In Fig. 2*A*, the nutrients are well-mixed ($\tau_{D}=0$), and all 10 species coexist at steady state. The steady state of the spatial case shown in Fig. 2*B* still exceeds competitive exclusion, with 3 species coexisting on 2 resources, but much of the biodiversity is lost. This behavior is striking, as it contrasts with many competition models where spatial structure increases diversity relative to the well-mixed case (20–26). In those models, diversity increases because spatial segregation, combined with local interactions, weakens interspecific competition. Here, however, the resource environment is uniformly coupled via diffusion, so competition remains strong. Strategies that are poorly matched to the nutrient supply allow unused nutrients to diffuse away to competitors; such populations shrink until the nutrient fluxes are balanced or the species goes extinct. This contrasts with the well-mixed case, where at steady state all of the nutrient concentrations are equal so that every strategy can coexist (8). Thus, territorial spatial structure heightens competitive differences between strategies, even when all obey the same trade-offs.

How representative is the behavior seen in Fig. 2 *A* and *B*? In Fig. 2 *C* and *D*, we show results for many randomly generated territorial communities, confirming that the loss of biodiversity is a generic feature of spatial structure and that the nutrient diffusion time $\tau_{D}$ acts as a control parameter for biodiversity. In the well-mixed model, all 10 species typically coexist. (See ref. 8 for a discussion of the “convex hull condition” for coexistence.) Fig. 2*C* shows the mean fraction of species coexisting at steady state for nonzero $\tau_{D}$. Spatial communities still violate competitive exclusion, but a large fraction of species go extinct. Even among those that survive, spatial structure reduces biodiversity by rendering abundances highly unequal. Using the same data as Fig. 2*C*, Fig. 2*D* quantifies this via the effective number of species M=exp(H), where $H=-\sum_{\sigma }p_{\sigma }\log p_{\sigma }$ is the Shannon entropy and $p_{\sigma }=n_{\sigma }/L$. (Intuitively, M is the number of equal populations yielding H. See *SI Appendix* for full rank abundance curves.) The average community loses ≈ 1/3 of its steady-state diversity as $\tau_{D}$ grows from 0.01 to 1600. In well-mixed communities, all 10 species are typically present in comparable proportions, but in the spatial model, 1 species dominates. Once this population is large compared to $\sqrt{E/D}$, increasing $\tau_{D}$ adds to the “bulk” population in its interior, decreasing overall diversity (see *SI Appendix* for details). However, aggregate measures of diversity in Fig. 2 *C* and *D* belies a wide distribution of outcomes. For example, only 3 species survive in Fig. 2*B*, whereas 9 coexist in Fig. 1. Why are some steady-state communities so much more diverse than others?

**Fig. 3.**
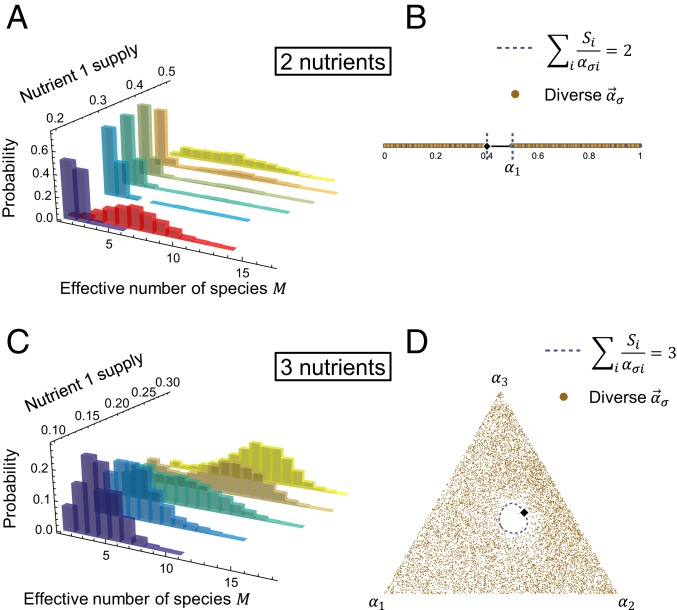
Steady-state diversity is governed by a simple condition: Diversity crashes if there is an “oligotroph” whose strategy satisfies $R_{\sigma }<p$. (*A*) Probability of effective number of species M at steady state. For each nutrient supply, we simulated 2,000 sets of 20 strategies. Strategies were chosen uniformly at random, except the case shown in red ($\vec{s}=\left(0.2,0.8\right)$), where oligotrophs were excluded. $\tau_{D}=10$ here and below. (*B*) For $\vec{s}=\left(0.4,0.6\right)$ (orange in *A*), we plot all strategies that appear in the most diverse 10% of simulations (90th percentile and above of M). No strategies appear in the oligotroph region, demarcated by the blue dashed lines. (*C*) Same as *A* but for 3 nutrients. (*D*) Strategies that appear in the most diverse 10% of simulations for $\vec{s}=\left(0.2,0.4,0.4\right)$ (teal in *C*). The oligotroph region is nearly empty.

In order to identify which features of the initial set of species determine steady-state diversity, we generated many random communities with species drawn uniformly from strategy space. Fig. 3 *A* and *C* shows the distributions of the steady-state diversity M as a function of $s_{1}$, the supply of nutrient 1. The number of nutrients does not explain the difference in outcomes. However, diverse steady states proliferate as the supply becomes more balanced between nutrients. What distinguishes high-diversity outcomes? Fig. 3 *B* and *D* shows every strategy present in every community with high diversity. Diverse communities have one thing in common: They lack species in the region of strategy space where $R_{\sigma }\equiv \sum_{i}^{p}R_{\sigma i}<p$. Here, $R_{\sigma i}\equiv S_{i}/\alpha_{\sigma i}$ is the uniform concentration of nutrient i an isolated population with uptake $\alpha_{\sigma i}$ would produce given a supply rate $S_{i}$. Thus, $R_{\sigma }$ is the total nutrient concentration maintained by and sustaining an isolated species σ at steady state. For comparison, $R_{\sigma }$ diverges for specialists ($\alpha_{\sigma i}=0$), while a perfect generalist ($\alpha_{\sigma i}=1/p$) has $R_{\sigma }=p$, as does a strategy that perfectly matches the supply ($\alpha_{\sigma i}=S_{i}$). Strategies satisfying $R_{\sigma }<p$ survive on even lower total nutrient concentrations, so we christen them “oligotrophs.” Their ability to create and survive on the minimum total nutrient concentration allows them to drive competitors extinct, thus reducing diversity. This recalls Tilman’s famous result that the species with the lowest equilibrium concentration of its limiting resource (the lowest $R^{*}$) can displace all others competing for that resource (15). However, the $R^{*}$ rule is due to a species’ innate superiority in consuming a single resource, whereas the oligotroph condition arises in a competition for multiple resources between intrinsically equal species.

To test whether it is simply the presence/absence of oligotrophs that controls overall biodiversity, we generated random communities in an environment with an asymmetric nutrient supply ($s_{1}=0.2$), but excluded oligotrophs. The resulting steady-state communities are much more diverse (Fig. 3*A*, red) than the case where oligotrophs are allowed (Fig. 3*A*, purple). Hence, an asymmetric nutrient supply reduces diversity by increasing the probability that an oligotroph will be present. The oligotroph condition captures the intuition of the $R^{*}$ rule—species with low resource requirements dominate—but does not require biological superiority or preclude coexistence beyond competitive exclusion.

### Alternative Steady States and Slow Dynamics.

How does the outcome of spatial competition depend on initial conditions? Consider the well-mixed system in Fig. 2*A*. All that differs between the top and bottom subplots are the initial populations, but the same set of species has 2 very different steady states; not even the hierarchy of populations is preserved. In fact, there is an m−p-dimensional degenerate manifold of fixed points corresponding to the communities that construct the same steady-state nutrient environment $c_{i}^{*}=S/E\;\forall \;i$. The final population may lie anywhere on this manifold. By contrast, in the spatial ecosystem of Fig. 2*B*, both sets of initial populations converge to the same unique steady state.

**Fig. 4.**
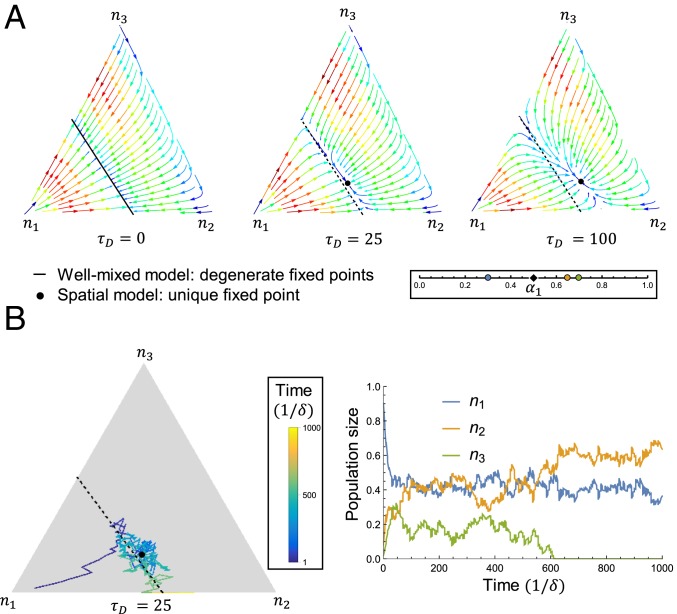
Spatial structure replaces the steady-state degeneracy of the well-mixed case with slow modes in population space. (*A*) Trajectories in population space for a 3-way competition at different values of $\tau_{D}$. The direction and color of the arrows show the direction and magnitude of $dn_{\sigma }/dt$, respectively. (*A*, *Inset*) Strategies and supply for *A* and *B*. (*B*) Same as *A*, but with stochastic dynamics due to random births and deaths; see *SI Appendix* for details. (*B*, *Inset*) Trajectory color as a function of time. (*B*, *Left*) Species 3 drifts to extinction. (*B*, *Right*) $n_{\sigma }\left(t\right)$ for the population trajectory at *B*, *Left*.

**Fig. 5.**
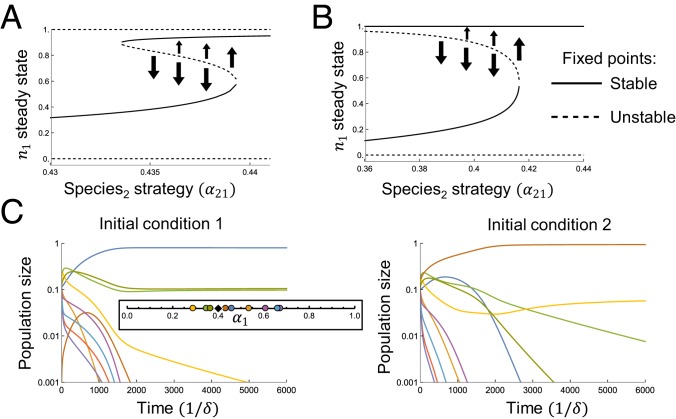
(*A* and *B*) Two species competing for 2 nutrients, with supply $\vec{s}=\left(0.3,0.7\right)$ and $\tau_{D}=400$. Arrows indicate flow away from unstable fixed points. (*A*) Bistability for $\alpha_{11}=0.29$. (*B*) Allee effect for $\alpha_{11}=0.31$. Species 2 goes extinct if its initial population is too low. (*C*) The Allee effect in a competition with 10 species and 2 nutrients, with $\vec{s}=\left(0.4,0.6\right)$ and $\tau_{D}=100$. The blue and brown species displace each other, depending on initial conditions. (*Inset*) Strategies and supply.

The relationship between the steady states in the well-mixed and spatial regimes can be visualized in a simple example. Fig. 4*A* shows the phase behavior of 3 species competing for 2 resources. Here, m−p=1, so the well-mixed case (Fig. 4 *A*, *Left*) has a 1-dimensional degeneracy of steady states. In the spatial community (Fig. 4 *A*, *Center* and *Right*), the degenerate manifold collapses to a single fixed point. (Here, the fixed point is unique, but this is not always the case; see Fig. 5.) This discontinuous change in the steady states is reflected in Fig. 2 *C* and *D*, where the diversity for any $\tau_{D}\;\neq \;0$ is substantially lower than for the well-mixed limit $\tau_{D}=0$. Fig. 4*A* also clarifies another striking difference between Fig. 2*A*, in which the well-mixed community approaches steady state at approximately the individual death rate δ, and Fig. 2*B*, in which the spatial community approaches steady-state orders of magnitude more slowly. This emergent slow timescale and the breaking of degeneracy are intimately related: For any nonzero diffusion time $\tau_{D}$, the degenerate manifold becomes a corresponding slow manifold, which the population rapidly reaches and then crawls to a fixed point. Linear stability analysis around this fixed point reveals a relaxation time $t_{\text{slow}}∼1/\tau_{D}$, which diverges as $\tau_{D}\to 0$. (See *SI Appendix* for details.)

What are the ecological implications of this slow relaxation to steady state? In general, diverse communities with m−p ≫ 1 could have tens or hundreds of slow modes for population changes. These modes shape the response to perturbations: A microbial community might recover from one antibiotic very rapidly and another very slowly, depending on the shift in population space. Even without an intervention, real populations will have stochastic fluctuations around the steady state. Fig. 4*B* shows trajectories through population space for the same species and nutrient supply as in Fig. 4*A*, but with demographic noise due to stochastic births and deaths. Ecological drift is confined to the slow manifold, and fluctuations primarily excite the population’s “soft mode” of the balance between species 2 and species 3. These 2 have similar strategies, and either can drift to extinction, whereas species 1 always survives. In the absence of noise, increasing $\tau_{D}$ decreases fixed-point diversity (Fig. 2*D*). With noise, however, steady states in the well-mixed limit are unstable to fluctuations along the degenerate manifold. Increasing the “restoring force” ($∼\tau_{D}$) can prevent species from fluctuating to extinction, and so spatial structure can stabilize diversity.

Although the well-mixed case has degenerate steady states, the steady-state nutrient environment is unique, and small initial population differences lead to small differences in the steady state (Fig. 4*A*). By contrast, spatial communities can have multiple steady-state nutrient environments, and similar populations may end in very different steady states. For example, in a competition of 2 species for 2 resources, Fig. 5 *A* and *B* shows the steady states as a function of $\alpha_{21}$, with $\alpha_{11}$ held fixed. ($\alpha_{\sigma 1}$ is the enzyme allocation of species σ to nutrient 1. Due to trade-offs, this also fixes $\alpha_{\sigma 2}$.) In Fig. 5*A*, there are 2 alternative steady states with both species coexisting. The unstable fixed point separates the relatively equal community of the lower branch from the upper branch, where species 1 dominates. This bistability leads to discontinuous transitions, where small changes (the populations crossing the separatrix, or the strategy exiting the bistable phase) can have dramatic consequences. Fig. 5*B* shows another region of strategy space where the outcomes are bistable, but now the alternatives are coexistence and exclusion. Above the unstable fixed point in Fig. 5*B*, species 1 drives species 2 to extinction. Otherwise, they coexist with species 2 having the larger population. This is an example of the Allee effect: Species 2 can only survive if its population exceeds a threshold. (See *SI Appendix* for a phase diagram of the full strategy space.)

The Allee effect persists in more complex communities. Fig. 5*C* shows a 10-species competition where the brown and blue species can displace each other depending on the initial conditions, modifying the 8 other species’ fates in the process. Thus, multistability and the Allee effect emerge naturally in our territorial model, even though the species interact exclusively through competition for resources.

### Unequal Enzyme Budgets.

**Fig. 6.**
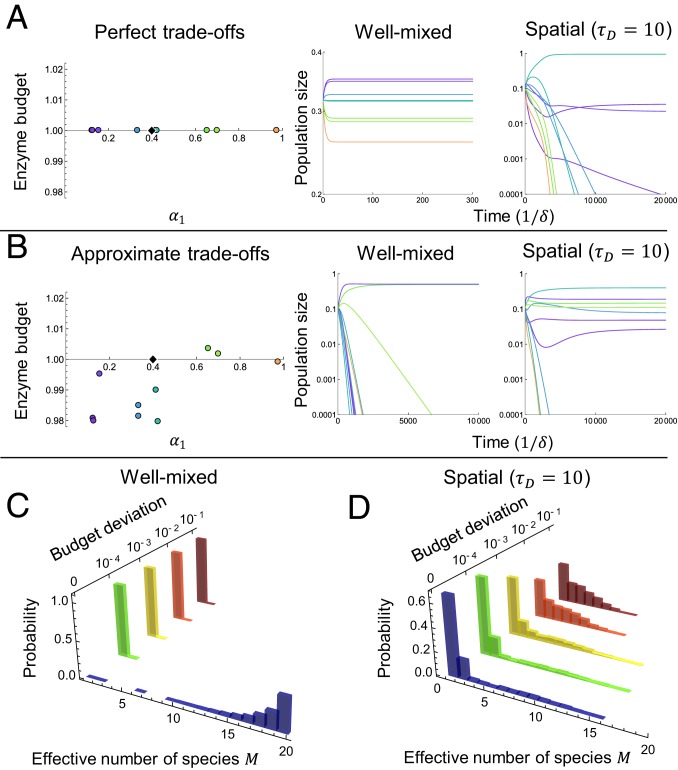
Territorial spatial structure renders diversity robust to variation in enzyme budgets. (*A*) In a community with equal enzyme budgets and $\vec{s}=\left(0.4,0.6\right)$ (*Left*), 10 species coexist in the well-mixed model (*Center*), whereas only 3 coexist in the spatial model (*Right*). (*B*) In a community with the same strategies as *A* but unequal enzyme budgets (*Left*), 2 species coexist in the well-mixed model (*Center*), whereas 7 coexist in the spatial model (*Right*). (*C*) Probability of effective number of species M at steady state for random enzyme budgets, in the well-mixed model with $\vec{s}=\left(0.4,0.6\right)$. For 2,000 sets of strategies, each of 20 initial species’ enzyme budgets $E_{\sigma }$ was drawn from N(1,δE). (*D*) Same as *C* but for spatial model.

Metabolic trade-offs are plausible because all microbes face the same biophysical constraints on metabolism and protein production, but trade-offs are unlikely to be exact in real ecosystems. How does this impact biodiversity in our model? Fig. 6*A* shows results for 10 species with exact trade-offs ($\sum_{i}\alpha_{\sigma i}=E$ for all species) competing for 2 resources. The well-mixed community is very diverse, while the spatial community is not. In Fig. 6*B*, each species allocates the same fraction of its enzyme budget to each nutrient as in Fig. 6*A*, but each with its own total enzyme budget $E_{\sigma }$. Diversity collapses in the well-mixed system, but the spatial community actually becomes more diverse. Fig. 6 *C* and *D* shows that this behavior is typical via comparison of the steady-state diversity of communities where each species’ enzyme budget is drawn from a normal distribution with mean 1 and SD δE. Well-mixed communities (Fig. 6*C*) are very diverse if trade-offs are exact (δE=0), but any disparity in the enzyme budgets causes diversity to collapse; only 1 or 2 species survives at steady state. The spatial communities (Fig. 6*D*) are less diverse for δE=0, but their diversity ⟨M⟩ actually increases with δE. This is due to an asymmetric effect: Oligotrophs lose their dominance with a very small decrease in $E_{\sigma }$, but other species require a large increase in $E_{\sigma }$ to dominate (see *SI Appendix* for details). As a result, spatial communities with imperfect trade-offs can display biodiversity well beyond the competitive-exclusion limit.

## Discussion

We analyzed a model of spatial resource competition among territorial surface communities such as biofilms, vegetation, or coral. Each species has a concrete metabolic strategy subject to biophysical trade-offs. The nutrient environment has no intrinsic heterogeneity but is globally coupled via diffusion, so competitors shape it via consumption. We found that the resulting spatial structure restricts biodiversity, in stark contrast to previous models, where spatial segregation increases diversity by weakening competition. In the simplest of these cases, different resources are partitioned into different regions, providing spatial niches (9–13). Alternatively, competitors may self-organize into patches linked by migration (14–16, 18, 19) or into aggregates with local interactions (20–26). External resource gradients can also increase diversity, because diffusion of dense motile populations prevents any species from monopolizing resource-rich regions (9, 10). In our model, the situation is very different. Because the external nutrient supply is uniform and the entire space is linked via diffusion, no spatial niches emerge; competitors have nowhere to hide. This is reminiscent of ecological reaction–diffusion models without external sources, where global coupling reduces diversity (26) and nonuniform steady states only become possible for unequal diffusion coefficients or complex geometries (27). Our communities are fundamentally different, however, as they occupy exclusive territories and exceed the competitive-exclusion limit despite a simple geometry and uniform diffusion coefficients.

What controls diversity in our model? The degree of nutrient mixing $\tau_{D}$ controls the evenness of abundances by setting the population of the dominant species, while the presence of oligotrophs distinguishes steady states of high coexistence from those with many extinctions. Oligotrophs drive competitors extinct because they have the lowest total nutrient requirements, in rough analogy with the lowest $R^{*}$ rule for well-mixed systems (15). However, oligotrophs obey the same trade-offs as every other species, and their dominance arises from the relationship between their strategies and the nutrient supply rather than any innate superiority. The composition of the nutrient supply sets the strategy range of oligotrophs, so it is effectively another control parameter for diversity. Despite highly nonlinear dynamics and many parameters, the oligotroph condition provides a simple criterion for diversity.

Spatial structure also provides a novel mechanism for discontinuous transitions between alternative steady states. Such sudden shifts, or “catastrophes,” attract significant attention due to their implications for ecosystem resilience (31). The Allee effect occurs in a large variety of ecosystems (32) and is particularly relevant to the conservation of rare species. It is usually understood as the result of transparently cooperative processes, such as production of a public good (32), and modeled via an explicit cooperative term. In resource-competition models, multistability has been observed when species consume nutrients one at a time (33) or with unequal stoichiometries (34). Here, both the Allee effect and multistability emerge naturally from the ability of a population to render its resource environment more favorable to itself. Interestingly, the Allee-effect species are oligotrophs, underscoring the special ability these strategies have to impact their ecosystems.

It has been observed that spatial structure increases the time to reach equilibrium (35). Here, we showed precisely how a new dynamical timescale emerges from spatial structure. We found that the slow dynamics are confined to a manifold in population space. These slow modes of the population are subject to large fluctuations due to noise (e.g., demographics). Slow relaxation also means that for a rapidly changing nutrient supply, the population might never reach steady state, potentially saving some species from extinction.

Finally, we find that spatial structure allows diversity to persist with imprecise metabolic trade-offs. In the well-mixed system without noise, any deviation from exactly equal enzyme budgets leads to ecosystem collapse (8). Spatial communities, however, remain diverse with only approximate trade-offs. In fact, variation in enzyme budgets actually increases mean diversity by impairing oligotrophs. The persistence of diversity beyond competitive exclusion with inexact trade-offs makes it more credible that trade-offs play a role in maintaining the surprising diversity of real ecosystems.

Our results suggest several future research directions. A 2D extension of the model exhibits the same loss of biodiversity due to oligotrophs and uneven abundances (*SI Appendix*), and it will be interesting to explore 2D pattern formation in more depth. One might also consider resources that diffuse at different rates. This can lead to nonuniform steady states in reaction–diffusion systems (27). Finally, in microbial communities, gene regulation and evolution are often relevant on ecological timescales, so it would be natural to allow species to modify their strategies.

In summary, we find that spatial structure engenders more realistic communities: It curtails the unlimited diversity of the well-mixed model, but allows for coexistence beyond the competitive exclusion principle even in the absence of exact metabolic trade-offs. Our results demonstrate that mechanistic interactions, arising from biophysical constraints such as space and metabolism, can allow even simple models to capture some of the rich behaviors of real ecosystems.

## Materials and Methods

The population ordinary differential equations (Eq. **3**) were solved numerically by using Mathematica’s “NDSolve.” The $c_{\sigma ,i}$ depend on $n_{\sigma }$ through the coefficients $\left\{A_{\sigma i},B_{\sigma i}\right\}$, which are fixed by requiring that $c_{i}\left(x\right)$ be continuous and differentiable at the population boundaries. The nutrient equations are simpler under the change of variables $x\to x-\underset{\sigma \prime <\sigma }{\sum }\;n_{\sigma \prime }$. Then, $c_{\sigma ,i}\left(x\right)$ runs from 0 to $n_{\sigma }$, yielding the system

$$\begin{array}{cc} c_{\sigma ,i}\left(n_{\sigma }\right) & =c_{\sigma +1,i}\left(0\right)\\ c_{\sigma ,i}^{\prime }\left(n_{\sigma }\right) & =c_{\sigma +1,i}^{\prime }\left(0\right), \end{array}$$ [4]

which was solved by using Mathematica’s “LinearSolve” with periodic boundary conditions ($c_{mi}\left(n_{m}\right)=c_{1i}\left(0\right)$, corresponding to a ring).

Details on the well-mixed model, stochastic dynamics, and figure parameters can be found in *SI Appendix*. Code is available on GitHub (36).

## Supplementary Material

Supplementary File
